# Synergistic Thermal Enhancement of Embedded Micro-Pyramid Array and Advanced Nanofluids for High Heat Dissipation

**DOI:** 10.3390/mi17040410

**Published:** 2026-03-27

**Authors:** Yafan Qin, Jingtan Chen, Xing Yang, Yuefei Yan, Shikun Zheng, Xiaofei Ma, Meng Wang, Congsi Wang

**Affiliations:** 1 State Key Laboratory of Electromechanical Integrated Manufacturing of High-Performance Electronic Equipments, Xidian University, Xi’an 710071, China; yafanqin@stu.xidian.edu.cn (Y.Q.);; 2 School of Mechano-Electronic Engineering, Xidian University, Xi’an 710071, China; 3 Guangzhou Institute of Technology, Xidian University, Guangzhou 510555, China; 4 College of Mechanical and Electrical Engineering, Shaanxi University of Science and Technology, Xi’an 710021, China; 5 Xi’an Institute of Space Radio Technology, Xi’an 710100, China; 6 Shaanxi Huanghe Group Co., Ltd., Xi’an 710043, China

**Keywords:** embedded cooling, micro-pyramid array, nanofluid, structural optimization, heat dissipation enhancement

## Abstract

The escalating power density in Active Phased Array Radar has made the thermal management of Transmitter and Receiver (T/R) modules a critical bottleneck for radar performance. To address the thermal resistance of traditional cold plates, this study investigates an innovative embedded cooling strategy utilizing micro-pyramid arrays and advanced nanofluids. Thermal performance was evaluated using maximum temperature, maximum temperature difference and surface temperature standard deviation (ST). Higher pyramid density markedly enhances temperature uniformity, an effect that scales positively with the power load. Under a 100 W condition, the 8-circle micro-pyramids configuration (the densest structure with roughness Ra = 1.3) achieved a 22.58 K reduction in maximum temperature and a 22.5% improvement in temperature uniformity compared to the 2-circle structure, and outperformed the 4-circle structure by 16.98 K and 17.9%, respectively. Furthermore, a comparative analysis of nanofluids (Al_2_O_3_, CuO, graphene, and h-BN) is conducted and it is found that graphene nanofluid exhibits the best overall heat transfer enhancement because of its high thermal conductivity and moderate reduction in specific heat capacity. The thermal performance of the nanofluid is evaluated by comparing the maximum temperatures of the heat source at the 8-circle structure. The synergistic coupling of graphene nanofluid with the 8-circle array yields a remarkable 35.38% enhancement in temperature uniformity at 100 W. The enhancement mechanisms are mainly attributed to intrinsic thermophysical properties of the nanoparticles and convection caused by denser pyramid array. The aforementioned findings provide important guidance for the thermal management design of antenna and other high-density integrated electronic systems with embedded cold plate design demand.

## 1. Introduction

Active phased array antenna (APAA) opens up a broader space for radar development with advantages of antenna beam fast scanning, flexible change of beam shape, signal power spatial synthesis and other technical characteristics [1,2]. Therefore, it has been widely used in Beidou communication, star-carrying imaging, airborne warnings, intelligent vehicle-carried radar and other military and civil radar [3]. Transmitter/receiver (T/R) module is the main functional unit of radar and communication systems, responsible for signal transmission, reception, beam control and real-time processing. Its performance directly determines the detection accuracy, anti-interference ability and reliability, and it is the main unit of APAA and other modern electronic equipment to realize high efficiency and multi-functionality. The heat generation of T/R module accounts for about 85% of the total heat generation of the antenna [4]. High heat flux will cause the T/R module performance to temperature drift, resulting in a severe deterioration of electrical performance. It creates various problems, including heat concentration, limited heat dissipation area, diverse application conditions, complex and variable environment, interdisciplinary and multi-physical field coupling [5]. If effective heat dissipation cannot be carried out immediately, the increase of antenna array temperature will greatly affect the electromagnetic performance of the antenna, and even lead to problems such as unit failure [6]. Component thermal control technology and antenna performance enhancement complement each other, and the optimization of efficient heat dissipation solutions is imminent. The problem of antenna gain loss and sidelobe level elevation caused by high heat flux has become a key restriction in the implementation of major national defense strategies, such as alert reconnaissance, enemy and self-identification, auxiliary attack, precision guidance, and so on [7].

Currently, the commonly used methods in the field of electronic thermal management include air cooling, thermoelectric cooling [8], immersion cooling [9], phase change cooling [10], heat pipe cooling [11], spray cooling [12] and microchannel cooling [6]. The crucial goal is to reduce contact thermal resistance, improve temperature uniformity, reduce maximum temperature and eventually improve heat transfer efficiency [13]. Embedded cold plate can be directly integrated into the T/R module or tightly fit the heating element, without external cooling fins or complex pipes, with the help of the coolant’s (such as water or nano-fluid) high thermal conductivity, it can avoid the accumulation of thermal resistance problem of the traditional air-cooled or indirect heat dissipation [14,15]. Compared with traditional heat exchangers, the embedded liquid-cooled microchannel heat dissipation system not only has smaller size, larger heat transfer coefficient and higher heat transfer efficiency which can meet higher energy-efficiency standards, but also has lower contact thermal resistance.

Recently, there have been many experimental works on the thermal enhancement of embedded cold plate design. Nan Zhang et al. [16] used an on-chip internal enhanced cooling method where the embedded heat transfer structure was manufactured on the backside of the chip substrate. The study consisted of measuring and analyzing the chip surface temperature, the total thermal resistance and the pressure drop at different heating powers and flow rates. Because the design of the embedded cooling system is closely related to the chip structure [17], and the study of the embedded cooling structure is mostly analyzed by comparison of the cold plate temperature and pressure drop [18]. Ali et al. confirmed through comparative simulations that the combination of micro-pin fins with microchannels can effectively enhance the thermo-hydraulic performance of microchannel heat sinks; however, the resulting flow disturbance and convergence also lead to an increase in pressure drop [19]. Further, Zhao et al. investigated the relationship between variable-density micro pin-fins integrated with microchannels and temperature uniformity. Their results showed that strategically spaced variable-density pin-fins can enhance heat dissipation in regions with ultra-high heat flux hotspots while effectively maintaining a low increase in pressure drop [20]. Alam et al. examined the influence of the structural dimensions of tapered-angle pin fins on thermo-hydraulic performance. Their findings indicate that the optimal structural dimensions are correlated with flow rate: at low flow rates, a smaller taper angle is preferable, whereas as the flow rate increases, the optimal taper angle gradually becomes larger [21].

Zuyuan Wang et al. [22] investigated the thermal design of Printed Circuit Board (PCB) with nine different chips using micro pin-fin array heat sinks. Four types of micro pin-fin arrays were designed: the square-pin fin array, round-pin fin array, truncated-pyramid-pin fin array, and truncated-conical-pin fin array. The truncated-pyramid-pin fin array performed well in thermal management of the multi-chip PCB. Qifeng Zhu et al. [23] conducted a flow study of the micro-pyramid with different cross-sections, and the enhancement of the micro-pyramid for heat dissipation is mainly through changing the fluid distribution and flow trajectory, reducing the regional flow resistance and vortex. The study of fluid flow trajectory needs to be combined with Reynolds number for turbulence analysis and judgement, in order to determine the coolant flow rate. At the same time, some of the micro-pyramid structure innovation studies were conducted in parallel with the TSV encapsulation studies [24], which can be further analyzed for their engineering applicability. As the composite scales of microchannel structures have become more accurately defined, various innovations in the development of structural design have emerged, such as the use of bionic structures [25] and the optimization of the topology of the channels [26]. Sadri et al. [27] employed the differential transformation method (DTM) to derive and non-dimensionalize the general heat transfer equation for fins, revealing the effects of variations in thermophysical and geometric parameters on heat transfer performance. Babaelahi et al. [28] solved the nonlinear radiative fin heat transfer equation with temperature-dependent thermal conductivity using both the DTM and the optimal homotopy asymptotic method (OHAM), thereby theoretically elucidating the influence of thermal conductivity and geometric parameters on dimensionless temperature distribution and thermal efficiency. Bouaziz et al. [29] introduced a novel concept termed the double optimal linearization method (DOLM) and derived an analytical expression for the thermal performance of convective–radiative fins with temperature-dependent thermal conductivity, providing a theoretical tool for the thermal design and analysis of fin structures.

In summary, embedded cold plate designs have proven effective for dissipating high heat loads in electronic devices. However, as the heat flux rises to more than 1500 W/m^2^, the structure design of the embedded cold plate must be improved for the challenge. Currently, structures of embedded microchannels are not thoroughly studied and whether the conventional microchannel design structures are still effective under such extreme working conditions requires further evaluation.

Besides structure optimization, novel coolants are needed to further enhance the heat transfer efficiency. Hybrid nanofluids as a coolant can further enhance the heat exchange effect, and the magnetic or electrical conductivity properties of certain nanoparticles can also facilitate the transmission of electromagnetic signals [30]. Hamaza Babar et al. [31] conducted a bibliometric study of nanofluids and analyzed the types of nanofluids, heat transfer principles and the channel scales used. The more studied are Al_2_O_3_, CuO, TiO_2_, MgO and the commonly used solvents are ethylene glycol and deionized water. Experimental studies have further demonstrated that the thermal performance of Al_2_O_3_ and CuO nanofluids is strongly dependent on nanoparticle concentration, operating conditions, and suspension stability. For instance, Raveshi et al. [32] experimentally investigated the effect of alumina nanoparticles on the pool boiling heat transfer coefficient and reported that the nanoparticles significantly enhanced heat transfer, achieving a 64% improvement at a nanoparticle volume fraction of 0.75%. Zamzamian et al. [33] experimentally evaluated the influence of working temperature and particle concentration on the forced convective heat transfer coefficient of alumina/ethylene glycol and copper oxide/ethylene glycol nanofluids. Their results indicated an enhancement in the convective heat transfer coefficient ranging from 2% to 50% compared to the base fluid, with more pronounced improvements observed at higher temperatures and nanoparticle concentrations. Additionally, Heris et al. [34] systematically investigated the effects of nanoparticle volume fraction, flow velocity, and inlet temperature on the thermal performance of radiators using water/EG mixture-based nanofluids. The results demonstrated that, under optimal conditions, the Nusselt number (Nu) was enhanced by up to 55% compared to the base fluid. Literature studies are mostly combined with the effects from Reynolds number, showing that nanofluids have a large effect on temperature and pressure drop during heat dissipation. Researchers are continually revising the formulas for calculating the physical parameters and heat transfer characteristics of nanofluids in the studies of microchannel heat dissipation and fluid flow [35]. The microscopic study of nanofluids is mainly focused on the volume fraction, size and shape of nanoparticles [36] which maximize heat dissipation capacity. There have been studies using Al_2_O_3_, CuO and graphene nanofluids in microchannel coolers, which proved to be effective, but new nanoparticle, such as hexagonal boron nitride (BN) has not been extensively used in liquid-cooling research. Hence, to enhance the embedded cold plate performance, this work systematically compares the performance effects of different nanofluids, especially the advanced nanofluids on the proposed structures.

In this work, we construct a T/R module die model and micro-pyramids array embedded cold plate. The parameter effects of thermal design power (TDP) and different structures are simulated and the enhancement mechanisms are analyzed. Based on this, Al_2_O_3_, CuO, BN and graphene nanofluids are used as novel coolants to compare and analyze the heat dissipation performance through their maximum temperature, maximum temperature difference and heat transfer coefficient.

## 2. Model Description

### 2.1. Electronic Module Structure

**Figure 1:**
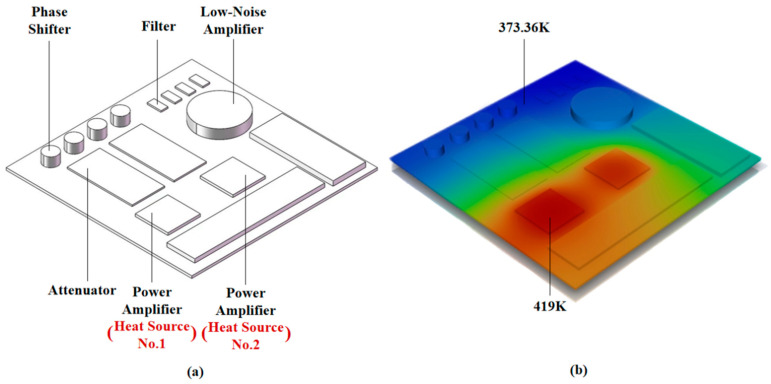
(**a**) T/R module 3D model and (**b**) surface temperature distribution diagram.

The T/R module mainly includes components such as Power Amplifier (PA), Low-Noise Amplifier (LNA), Phase Shifter, Attenuator, Filter, and so on, which are encapsulated in the module shell. The model diagram of the T/R module is shown in Figure 1a, which is drawn by SOLIDWORKS 2020 3D modelling software. The size of the base plate is 40 mm × 40 mm × 0.5 mm, two heat sources representing Power Amplifier (PA) are set, the size is 7 mm × 7 mm × 0.5 mm, and the maximum value of the *z*-axis direction of the model (the maximum thickness of the module) is 2.5 mm.

The base plate material is defined as aluminum carbide ceramic substrate, with the thermal conductivity of 30 W/(m·K), density of 3700 kg/m^3^, and specific heat capacity of 900 J/(kg·K); the heat source material is defined as aluminum. The simulation in this work focuses on the volume-to-surface (V-to-S) heat transfer problem. Equating the internally complex packaged modules to entities with uniform thermal properties is a widely adopted approach for simulation efficiency with acceptable accuracy [37,38]. The model parameters are shown in Table 1. The environment is simulated as low-velocity air forced convection, with an ambient temperature of 300 K and convective heat transfer coefficient of 60 W/(m^2^·K).

For the 40 mm scale type of device, a single heat source with a power of more than 7.5 W can be classified as a high-power device. In this paper, we set up two core elements with TDP of 10 W each. The two heat sources are equal in volume (24.5 mm^3^) and power (10 W, equivalent power density of 4 × 10^2^ W/cm^3^), and the heat sources are similarly arranged on the contact surface with the environment for convective heat transfer.

The standard deviation (*ST*) of the surface temperature of whole module is calculated for measuring the temperature uniformity of a multi-heat source component, and the calculation expression according to the Equation (1):

(1) $$ST=\sqrt{\frac{1}{n}\sum_{i=1}^{n}{\left(T_{i}-T_{ave}\right)}^{2}}$$

The temperature distribution diagram of T/R module under air convection condition is shown in Figure 1b. The maximum temperature of 419 K occurs at heat source No.1, while the minimum temperature of 373.36 K is observed at the filter units. The temperature distribution is asymmetric, where high temperature concentrated in the heat source point to the surrounding part of the dispersion, and the maximum temperature gradient is 45.64 K, standard deviation (ST) of the surface temperature is 23.138. The simulation results above indicate that the surface temperature of the T/R module increases sharply during operation. Excessive component temperatures can lead to thermal drift of electrical performance, while significant temperature differences may cause component deformation due to uneven thermal stress distribution. Thermal management design must be carried out to reduce the junction temperature of the power amplifiers, as well as the temperature differences.

### 2.2. Embedded Cold Plate Structure

Based on the comprehensive consideration of high heat flow density and electrical signal transmission, silicon adapter plate is adopted as the embedded cold plate material. Silicon adapter plate can achieve high-density transmission of electrical signals and high heat flow density heat dissipation, which is suitable for high-density integration of electronic microsystems with high thermal efficiency [39], and is also the hot spot of the current high-efficiency heat dissipation research [40].

A variety of micro-pyramids structures have appeared in the existing research, but they cause high levels of flow disturbance, flow resistance and pressure drop are relatively large [22]. Tapered micro-pyramids have been proven to reduce the momentum loss of the fluid, and maintain the heat transfer performance [18]. In this work, circular distributed micro-pyramid shape structure is designed to enhance the embedded cold plate thermal performance.

**Figure 2:**
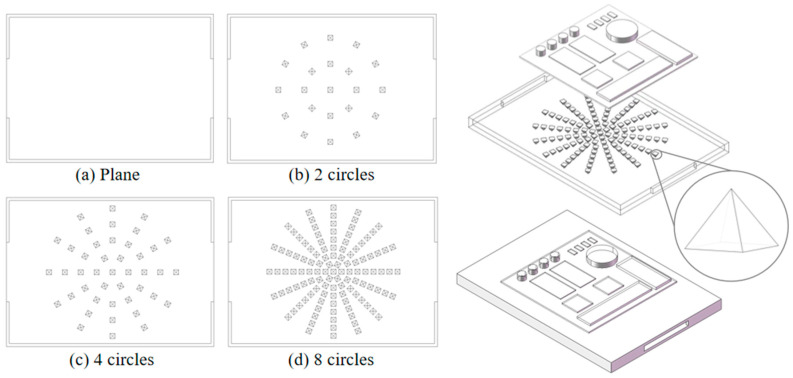
Cold plate structure and assembly diagram.

Figure 2 shows the cold plate structure and assembly model of the T/R module. The size of the cold plate box is 70 mm × 50 mm × 4 mm, with the inlet and outlet size of 20 mm × 2 mm. The size of the coolant flow area is 68 mm × 48 mm × 2 mm, the height of the flow channel is 2 mm. The thickness of the cold plate between the heat source and the coolant is 1 mm. The coolant flows from the left side to the right side, and the embedded cold plate is symmetrically designed. A buffer zone is designed to uniformly distribute fluid flow and reduce pressure drop fluctuations. Micro-pyramids using the bottom surface of a square four-pronged cone, four prongs and the bottom of the angle are 60°. The prism cone bottom side length of 1.68 mm, the height of 1.45 mm. Although the pyramids have millimeter-scale dimensions, they function as micro-scale surface features relative to the overall flow domain and are treated as surface roughness elements in our analysis.

The maximum diameter of the circular micro-pyramids array is 45 mm, and each layer of the circular array is equally spaced, which is divided into three groups according to the degree of sparsity and one group without structural design: group (a) is a planar structure without micro-pyramids array heat dissipation; group (b) has 2 circles in addition to the central structure, with 21 micro-pyramids arrays; group (c) has 4 circles in addition to the central structure, with 43 micro-pyramids arrays; and group (d) has 8 circles in addition to the central structure, with 115 micro-pyramids arrays.

The staggered circular array is used, and the structure of the micro-pyramids array is designed to be dense in the center, dispersed from the center to the surroundings, and sparser in the surroundings. The staggered distribution of the micro-pyramids array has better heat dissipation performance than the parallel distribution, and the staggered distribution can make the fluid distribution more uniform and less prone to abnormal pressure drop.

In the fluid physical property parameter setting, this paper adopts the pressure-based model solution to explore the impact of embedded liquid-cooled runner structure design on heat dissipation performance based on the energy equation and steady state calculation. The k-ω SST model with automatic Reynolds number adjustment is used. Grids change at the Reynolds number (Re) of 798.56 in the test, and the micro-pyramids array is not a microchannel in traditional significance, flow conditions and viscosity equations cannot be determined through simple Reynolds number calculations. Assumptions of incompressible fluid, constant flow, and no slip at fixed walls are applied in the fluid domain. The inlet velocity is set to 0.4 m/s and with a standard velocity-type inlet. The fluid inlet temperature used here is 315 K and the ambient temperature is 300 K. The outlet conditions are standard pressure type outlet and backflow suppression, and the gauge pressure is atmospheric pressure.

### 2.3. Grid Independent Verification and Model Validation

A grid independence test is performed to make sure that the solution is independent of the mesh refinement scheme. The optimal mesh number is determined and unified for different microchannel structure models. The geometric model is constructed in SOLIDWORKS, and then imported into the geometry module of ANSYS Workbench 2022. Boundary conditions is set and the built-in meshing tool is used to discretize the computational domain.

The solution process is configured in FLUENT. Pressure-based model applied in the model setup and the energy equation is activated. For air convection cooling simulation, the viscous model uses the k-epsilon realizable turbulence model with enhanced wall treatment is adopted for thermal boundary effects. Prior to simulation, the following assumptions are established: Incompressible fluid flow, Steady-state conditions and no-slip boundary condition at stationary walls. The working fluid is specified as incompressible air. The solution is iterated for 200 cycles, which is sufficient to achieve convergence with energy residual values below 10^−7^.

To reduce the influence of the solid area on the fluid area, the calculation domain we selected includes both the liquid and solid area in all simulation calculations. Comparing the test results, the relative error is calculated according to following Equation (2):

(2) $$e=\left|\frac{M_{2}-M_{1}}{M_{1}}\right|\times 100\%$$

where e represents relative error, M represents any parameter (maximum temperature is chosen in this article), $M_{1}$ represents the value of a parameter that is obtained from the finest grid, and $M_{2}$ represents the value of a parameter that is obtained from other grids.

**Figure 3:**
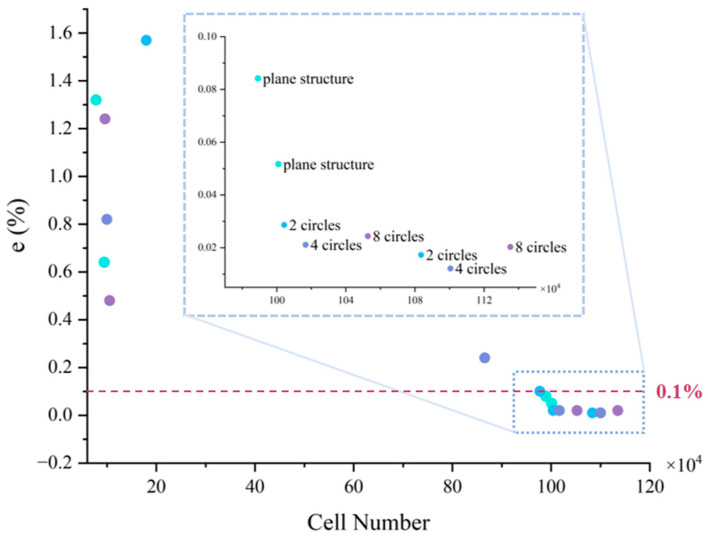
Grid independence validation: number of cells and error relation.

Figure 3 shows the relationship between the number of cells and the error (*e*), predominantly hexahedral elements with prism layers at the walls, mesh refinement was performed at a ratio of 1:1.5 in the heat source and its surrounding regions, at a ratio of 1:1.7 at the fluid-solid conjugate heat transfer interfaces, and at a ratio of 1:1.2 in the pyramid structures and their connecting regions. When the number of grid cells is 78,241 to 1,135,222. From the results, it is found that the temperature fluctuation tends to stabilize, the *e* is small and controllable within the range of 0.01% to 0.1%. Therefore, *e* under the ranges of 0.1% is selected, and the mesh scheme that ensures accuracy and also saves computational time is taken as shown in Table 2.

**Figure 4:**
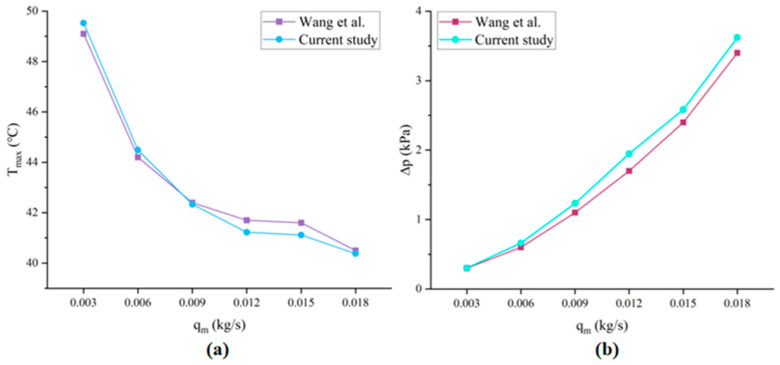
Comparison of the numerical simulation results of the proposed method in this study with the results of Wang et al. [22]. (**a**) Maximum temperature comparison; (**b**) Pressure drop comparison.

Subsequently, as shown in Figure 4, the experimental results of Wang et al. [22] are compared to validate the simulation model. The maximum temperature of the heat source and the inlet/outlet pressure drop are compared for quantitative analysis. The thermal performance of the truncated-pyramid-pin fin array heat sink is analyzed under conditions of inlet mass flow rates ranging from 0.003 kg/s to 0.018 kg/s and heat flux density of 30 W/cm^2^. By comparing with the experimental results, it can be observed that the simulation results show good agreement with the experimental data (within the error of 1.14% for temperature data and 5.53% for pressure data). The discrepancy is possibly caused by the simplification of geometric features and the idealization of boundary conditions. The validation is indirect but sufficient to establish confidence in our simulation methodology. Nevertheless, the current model successfully captures the thermal performance of embedded microchannels, and further analysis is presented in the next section.

### 2.4. Nanofluid Selection

In recent years, there have been many thermal enhancements adopting nanofluid for electronic heat dissipation [41]. Most of them focuses on nanoparticles such as Al_2_O_3_, CuO, TiO_2_, MgO, ZnO, and so on. Recently, there are many novel nanoparticles fabrication studies [10], and their thermal performance on embedded cold plate requires further analysis. This work compares four kinds of nanofluids for comparison, including Al_2_O_3_, CuO, hexagonal boron nitride (BN), and graphene. The nanofluids have a volume fraction of 5% (*φ* = 5%), the theoretical nanoparticle diameter of 40 nm [42], and the base fluid is selected as deionized water. The Al_2_O_3_ and CuO nanoparticles are spherical nanoparticles with the sphericity of 1, and the BN and graphene have a similar chemical structure, with the sphericity of 0.85. Footnotes are labeled in the following Table 3:

The nanofluid density is calculated as

(3) $$\rho_{nf}=\left(1-\varphi \right)\rho_{f}+\varphi \rho_{p}$$

where *φ* is the volume fraction of nanoparticles in the base fluid; *ρ_nf_* is the nanofluid density, *ρ_f_* is the base fluid density, and *ρ_P_* is the nanoparticle density.

The nanofluid specific heat capacity *C_nf_* is calculated as:

(4) $$C_{nf}=\frac{\left(1-\varphi \right){\left(\rho Cp\right)}_{f}+\varphi {\left(\rho Cp\right)}_{p}}{\left(1-\varphi \right)\rho_{f}+\varphi \rho_{p}}$$

The nanofluid thermal conductivity is calculated as:

(5) $$\frac{k_{nf}}{k_{f}}=k_{f}\left[\frac{k_{p}+\left(n-1\right)k_{f}-\left(n-1\right)\left(k_{f}-k_{p}\right)\varphi }{k_{p}+\left(n-1\right)k_{f}+\left(k_{f}-k_{p}\right)\varphi }\right]$$

where *k_nf_* is the nanofluid thermal conductivity, *k_f_* is the base fluid thermal conductivity, *k_p_* is the nanoparticle thermal conductivity; *n* is the shape function, The calculation formula is n=3/ψ, where *ψ* is the particle sphericity.

The empirical formula for calculating nanofluid viscosity is:

(6) $$\frac{\mu_{nf}}{\mu_{f}}=\left(1+373wt-85566wt^{2}\right){\left(\frac{T_{nf}}{T_{0}}\right)}^{0.4573}$$

where *wt* is the solution mass fraction (%) and *T*_0_ is the environmental temperature.

The four nanofluid parameters are shown in Table 4.

Nanofluid viscosity is calculated as

(7) $$\frac{\mu_{nf}}{\mu_{f}}=1.1065{\left(1+\frac{T_{nf}}{300}\right)}^{0.095}$$

The calculated density, specific heat capacity, thermal conductivity, and viscosity are input into the materials as fluids, and the materials of the fluids are set to the four nanofluids in the unit zone conditions.

## 3. Simulation Results Analysis

### 3.1. Effects of Structural Design on Thermal Performance

Four groups of structural design shown in Figure 2 are simulated under the heat source of 10 W. Preliminary results show that the maximum temperature is reduced by an average of 93.12 K and the maximum temperature difference is reduced by an average of 74% compared with the structure without embedded cold plate. To quantitatively analyze the effect of structure design on T/R module thermal performance, roughness is adopted to evaluate the surface structure.

The equation of roughness is shown in Equation (8), where $S_{a}$ is actual surface area, $S_{b}$ is projection surface area. The roughness of plane structure, 2 circles micro- pyramids, 4 circles micro-pyramids and 8 circles micro-pyramids is 1, 1.06, 1.11 and 1.3.

(8) $$Ra=\frac{S_{a}}{S_{b}}$$

**Figure 5:**
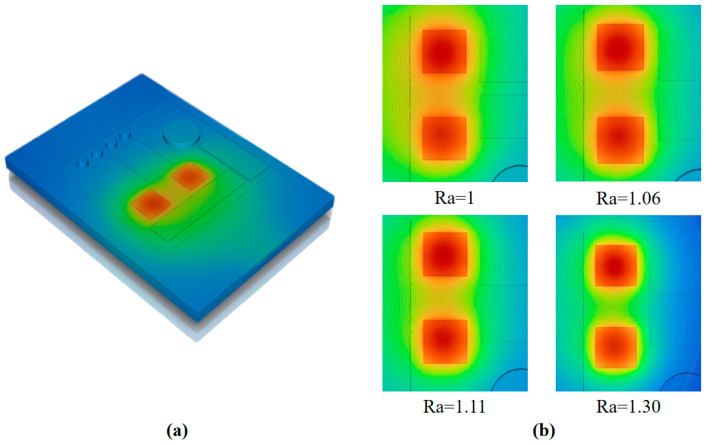
Temperature results at 10 W (**a**) Temperature contour of the chip with embedded cold plate; (**b**) Temperature distribution diagrams of the heat sources surroundings under different structures.

Results in Figure 5a show that temperature contour of the chip after embedded cold plate implementation. Compared to the non-cooled chip, the chip with the embedded cold plate exhibits significantly improved temperature uniformity. Figure 5b illustrates the temperature field distribution of the heat sources and their surroundings under different heat dissipation structures. It can be observed that the influence area of the heat source in the 8-circle structure is significantly smaller than that in the other three structures, and the issue of thermal overlap between the two heat sources is alleviated.

**Figure 6:**
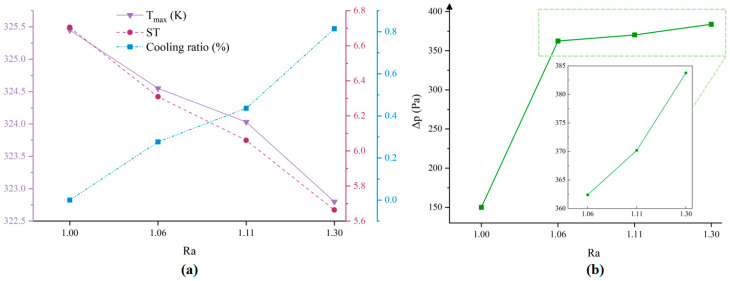
Temperature and pressure drop results for different structures under 10 W condition (**a**) Maximum temperature, maximum temperature difference, cooling ratio line chart; (**b**) Pressure drop results for different structures.

Figure 6a compares the thermal performance metrics (maximum temperature, standard deviation, cooling ratio) under different micro-pyramids arrays. Cooling ratio refers to the proportional reduction in maximum temperature, serving as a metric to quantify the extent of temperature decrease. Both maximum temperature and standard deviation decrease with structure densification, the reduction in temperature difference diminishes with the increment of surface roughness. Notably, the 8-circles micro-pyramids array configuration achieves a marked reduction in maximum temperature (1.23 K) but exhibits a more modest attenuation in standard deviation compared to 4-circles micro-pyramids array. This implies that the micro-pyramid arrays can simultaneously achieve maximum temperature suppression and optimized thermal field uniformity. However, the data indicate that a higher array density does not necessarily lead to a proportional improvement in heat dissipation performance. Figure 6b illustrates, and as the structure becomes denser, the pressure drop inevitably rises due to the flow disturbance caused by the staggered arrangement of the micro-pyramid structures. the pressure drop distribution of different structures. The incorporation of the pyramid structure significantly increases the inlet and outlet pressure drop of the microchannel.

In the development of the third generation of semiconductors, the degree of integration is constantly evolving. Therefore, research on ultra-high-power devices is necessary. The power of the heat source is set as 25 W, 50 W, 75 W and 100 W per heat source to further evaluating the thermal performance of the proposed structures.

**Figure 7:**
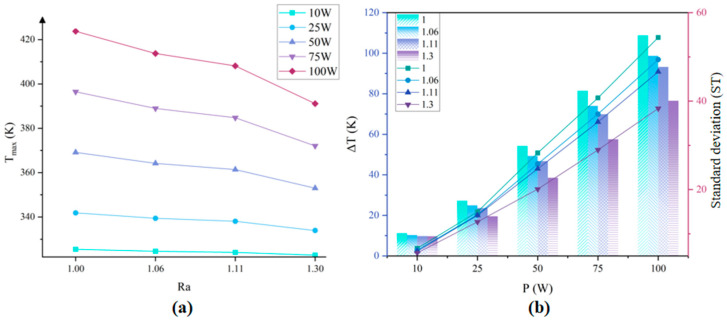
Effect of structural design on embedded cold plate thermal performance; (**a**) Maximum temperature under various heating power; (**b**) Standard deviation (ST) and maximum temperature difference under various heating power.

Figure 7a shows the variation in maximum temperature caused by structural densification under different power conditions. With the densification of the micro-pyramids array structure, the thermal enhancement performance varies. With the increment of heat source power, the reduction of maximum temperature increases with the increase of surface roughness. The maximum temperature of the chip is reduced by 4.97 K, 7.74 K, 16.18 K, and with the increase in the power of the heat source. It can be concluded that the structure densification provides better thermal enhancement under higher heat flux conditions.

Figure 7b shows the maximum temperature difference (bar chart) and standard deviation (*ST*) of the surface temperature (line graph) of different structural designs. The maximum temperature difference and its derived metrics are employed to assess the temperature uniformity of the thermal management system. While the maximum temperature difference inevitably increases with rising power input, the improvement in thermal uniformity induced by structural densification becomes more significant under same power conditions. The *ST* exhibits a decreasing trend with increasing roughness, and the difference becomes significant as power increases. Although the rising temperature in the heat source region leads to an increase in *ST* with higher power, it still demonstrates that structural densification can significantly improve temperature uniformity. Furthermore, the structure with the highest roughness demonstrates the greatest linear growth rate. Under the condition of 100 W per heat source, compared with the 4-circles micro-pyramids array structure, the 8-circles micro-pyramids array, the maximum temperature is reduced by 16.98 K, and the temperature uniformity is enhanced by 17.9%. Compared with the heat dissipation of 8 circles micro-pyramids array and 2 circles micro-pyramids array structure, the maximum temperature is reduced by 22.58 K, and the temperature uniformity is enhanced by 22.5%.

It can be seen that the densification of the micro-pyramids array has a significant impact on the heat dissipation effectiveness, and the thermal performance of the internal structure of the embedded microchannels becomes more significant as the electronic power level increases.

### 3.2. Effects of Working Fluids on Thermal Performance

In this section, devices with two 100 W heat sources each and an 8-circles micro-pyramids array structure are simulated for coolant effect analysis. Deionized water, with graphene exhibiting the most pronounced thermal improvement, followed by BN nanofluid. The Al_2_O_3_ and CuO nanofluids show comparable results. For the graphene nanofluid under 100 W operation with an 8-circles micro-pyramid array configuration, a 6.43 K reduction in maximum temperature and an 8.21% improvement in temperature uniformity are achieved.

A comprehensive comparison of the isobaric specific heat capacity (C_p_) and thermal conductivity (k) of these fluids relative to pure water reveals:

Specific heat capacity reductions: Graphene: 7.06%, BN: 8.83%, CuO: 21.80%, Al_2_O_3_: 13.66%.

Thermal conductivity enhancements: Graphene: 27.53%, BN: 21.26%, Al_2_O_3_: 7.41%, CuO: 8.25%.

The results indicate that the heat transfer enhancement performance of nanofluids is a competing outcome of increased thermal conductivity and reduced specific heat capacity. Although the significant improvement in k is the dominant factor for performance enhancement, the simple arithmetic difference between the changes in k and Cp is insufficient to accurately evaluate their overall heat transfer capability [43]. Under the conditions of this study, graphene and BN nanofluids exhibit the highest thermal conductivity enhancement efficiency and a relatively favorable combination of properties, showing the greatest application potential.

**Figure 8:**
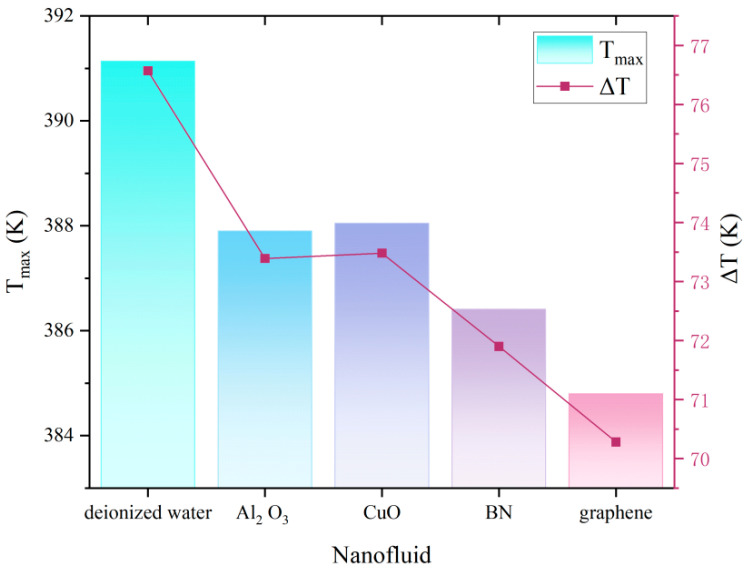
Effect of nanoparticle material on embedded cold plate maximum temperature performance.

As shown in Figure 8, all four nanofluids exhibit significant heat dissipation enhancement effects. Graphene achieves optimal heat dissipation due to its exceptionally high thermal conductivity and relatively minor reduction in specific heat capacity, synergistically maximizing convective heat transfer efficiency. The integrated nanofluid and structural design strategy achieves optimal heat transfer enhancement under two heat sources each of 100 W: the 8-circles micro-pyramid array with graphene nanofluid achieves a 38.96 K reduction in maximum temperature and 35.38% enhancement in temperature uniformity compared to the plane structure.

The above results prove that nanofluids have an obvious strengthening effect on heat dissipation, future work needs to systematically analyze various nanofluid variables (mass concentration, nanoparticle size and sedimentation behavior) on the embedded cold plate thermal enhancement.

### 3.3. Thermal Enhancement Mechanism of Circular Micro-Pyramids Array Structure

The previous section compares simulation results across 10 W, 25 W, 50 W, 75 W, and 100 W per heat source, demonstrating that structural densification of the micro-pyramids array enhances the heat dissipation capability of the embedded cold plate. To further investigate the embedded cooling enhancement induced by structural densification, a comparative analysis is conducted by introducing the heat transfer coefficient (HTC), which holistically evaluates conductive and convective heat transfer performance. The heat transfer coefficient is defined as the proportionality constant between the heat flux (the rate of heat transfer per unit area) and the thermodynamic driving force for heat flow, which is the temperature difference between the surface and the fluid. Mathematically, it can be expressed as the Equation (9):

(9) $$H=\frac{q}{T_{s}-T_{f}}$$

where *h* is the heat transfer coefficient, *q* is the thermal power per unit area, *T_s_* is the surface temperature. The undersurface of the body heat source is selected here, which is the contact surface between the heat source and the embedded cold plate. And *T_f_* is the surrounding fluid area temperature. The bottom surface of the embedded cold plate in contact with the fluid is selected here, which is the fluid-solid interface away from the heat source.

**Figure 9:**
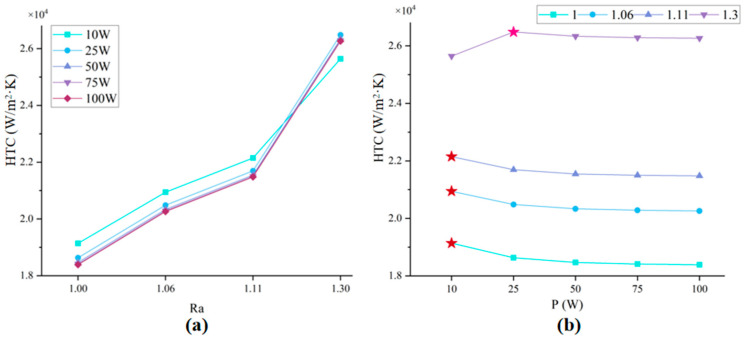
Results analysis of HTC (**a**) effect of structure roughness on HTC enhancement; (**b**) effect of input power on HTC performance.

Figure 9a shows that the HTC trends under five power levels with respect to structural variations. For given power input, the HTC exhibits a significant upward trend with progressive structural densification, indicating the thermal enhancement effect of micro-pyramids array densification. Notably, 10 W condition achieves the optimal overall heat dissipation performance, however, the incremental improvement from 4-circles to 8-circles configurations is significantly smaller compared to higher power cases, increasing only by 3.5 × 10^3^, while the other structures increased by approximately 5 × 10^3^. In contrast, the remaining four power conditions (25 W–100 W per heat source) exhibit a slight decline in thermal dissipation performance as power increases.

Figure 9b shows that under conditions with Ra < 1.3, the HTC monotonically decreases with increasing TDP, reaching peak performance at 10 W per heat source. This indicates that Ra < 1.3 structural configurations exhibit insufficient thermal dissipation capacity beyond power regimes, leading to performance degradation. Remarkably, the densest micro-pyramid array (8-circles) shifts its optimal operating power from 10 W to 25 W per heat source, as indicated by the star marks, only the highest HTC value for the eight-circle structure occurs at a heating power of 25 W, demonstrating enhanced thermal dissipation capability. A distinct performance peak emerges at 25 W per heat source, suggesting that progressive structural densification raises the critical power threshold for optimal operation.

The decline in HTC with increasing power is likely attributable to material-limited heat dissipation capacity, where thermal transport approaches the intrinsic conductive and convective thresholds of the substrate material. Therefore, selecting appropriate coolants can further enhance heat extraction efficiency, potentially overcoming material thermal limitations and optimizing overall system performance.

Under same power conditions, structural densification of the micro-pyramids array leads to a significant enhancement in heat dissipation performance. Each configuration has its unique peak heat dissipation capacity, while distinct power levels correspond to optimally efficient structural designs. These findings have significant implications for guiding structural design principles, particularly in achieving roughness-optimal power matching for maximized thermal efficiency in high-density electronic systems.

## 4. Conclusions

With increasing integration levels of electronic equipment, thermal management requirements have become more stringent. As the key component of APAA, T/R modules demand advanced cooling solutions. Embedded cold plate technology, which directly integrates cooling channels at critical heat source locations, achieves reduced contact thermal resistance and enhanced heat dissipation efficiency. This study focuses on microchannel design optimization for T/R modules based on embedded liquid cooling technology, further improving thermal performance through the incorporation of nanofluids. Below are main conclusions of this work:

1. T/R modules with a TDP of 10 W each exhibits asymmetric temperature field, with the maximum temperature reaching 419 K, maximum temperature difference of 45.64 K and standard deviation (ST) of the surface temperature is 23.138, highlighting the critical need for efficient thermal management solutions.
2. Four embedded cold plates are designed to enhance heat dissipation. Under two heat sources each of 10 W conditions, structural densification leads to improvements in maximum temperature, maximum temperature difference, and cooling ratio. The 8-circles micro-pyramids array reduces the maximum temperature by 1.23 K compared to the plane structure, but the enhancement is relatively minor.
3. The TDP of the T/R modules is elevated to 25 W, 50 W, 75 W, and 100 W per heat source, further validating the thermal performance enhancement from structural densification. At 100 W per heat source, the 8-circles micro-pyramids array, compared to the 2-circles configuration, achieves 22.58 K reduction in maximum temperature and 22.5% enhancement in temperature uniformity, highlighting the scalability of densified architectures under extreme thermal loads.
4. Four types of nanoparticles were chosen: Al_2_O_3_, CuO, graphene, and hexagonal BN, mixed with deionized water at a 5% volume fraction as coolants. The degree of heat dissipation enhancement is graphene-H_2_O > BN-H_2_O > Al_2_O_3_-H_2_O > CuO-H_2_O.
5. The HTC is significantly enhanced by structural densification under the same power conditions. For plane, 2-circles, and 4-circles structure, HTC shows decline trend with increasing power inputs. In contrast, the 8-circles structure’s HTC initially rises with increasing power but subsequently decreases beyond a critical threshold. Each configuration has its unique peak heat dissipation capacity, and the optimal structural density for maximum cooling efficiency varies with thermal load.

## Figures and Tables

**Table 1:** T/R module model parameters.

Component	Materials	Dimension (mm)	Density ρ/(kg·m^−3^)	Specific Heat Capacity C_p_/(J·kg^−1^·K^−1^)	Thermal Conductivity k/(W·m^−1^·K^−1^)
Base plate	Alumina ceramics substrate	40 × 40 × 0.5	3700	900	30
Heat Source No.1, 2	aluminum	7 × 7 × 0.5	2719	871	202.4
Other components	aluminum	-	2719	871	202.4

**Table 2:** Number of grid nodes and cells.

Structure	Number of Nodes	Number of Cells	*e* (%)
plane structure	4,270,169	989,156	0.08
2 circles	4,323,710	1,004,835	0.02
4 circles	4,475,919	1,016,697	0.02
8 circles	4,580,007	1,052,713	0.04

**Table 3:** The corresponding meanings of footnotes.

Footnote	Meaning
*nf*	nanofluid
*f*	base fluid
*P*	nanoparticle

**Table 4:** Calculated thermophysical properties of nanofluids at 315 K.

Nanofluid Type	Density ρ/(kg·m^−3^)	Specific Heat Capacity cp/(J·kg^−1^·K^−1^)	Thermal Conductivity k/(W·m^−1^·K^−1^)
Al_2_O_3_-H_2_O	1148.5	3626.19	0.648
CuO-H_2_O	1266	3484.40	0.654
BN-H_2_O	1062.5	3829	0.762
graphene-H_2_O	1062.5	3903.53	0.828

## Data Availability

The original contributions presented in this study are included in the article. Further inquiries can be directed to the corresponding authors.

## References

[1] Wang C., Yuan S., Gao W., Jiang C., Yan Y., Zheng Y., Wang Z., Wang M., Song X. (2021). A Prior Knowledge-Based Algorithm for Robust Design of Array Antennas With Interval Excitation and Position Uncertainties. IEEE Trans. Antennas Propagat..

[2] Wang C., Wang Y., Chen Y., Gao W., Xu Q., Wang Z., Liu J., Zhou C., Xu W., Zhong J. (2020). Coupling Model and Electronic Compensation of Antenna-Radome System for Hypersonic Vehicle With Effect of High-Temperature Ablation. IEEE Trans. Antennas Propagat..

[3] Lian P., Wang C., Xue S., Wang Y., Yan Y., Xu Q., Duan B., Wang N., Duan Y., Wu Y. (2021). Future Research Trend for Improving Large Reflector Antenna Service Performance. Engineering.

[4] Wang Y., Wang C., Lian P., Xue S., Liu J., Gao W., Shi Y., Wang Z., Yu K., Peng X. (2020). Effect of Temperature on Electromagnetic Performance of Active Phased Array Antenna. Electronics.

[5] Xu P., Lin C., Han B., Wang Z., Yu K., Yin K., Leng G., Wang Y., Li Z., Ma X. (2022). Electromagnetic-Structural-Thermal Coupling Theory for Array Antenna_ Present and Future. Acta Electron. Sin..

[6] Yang X., Wang Y., Yan Y., Chen J., Tan J., Wang X., Du B., Zhang J., Wang M., Tao Y. (2024). Establishment and assessment of a variable-area parameterized model of microchannel for high temperature uniformity. Appl. Therm. Eng..

[7] Wang C., Han R., Wang W., Wang M., Tu Q., Ping L. (2016). Development of Spaceborne Deployable Active Phased Array Antennas. J. Mech. Eng..

[8] Singh V.K., Sisodia S.S., Patel A., Shah T., Das P., Patel R.N., Bhavsar R.R. (2023). Thermoelectric cooler (TEC) based thermal control system for space applications: Numerical study. Appl. Therm. Eng..

[9] Pambudi N.A., Sarifudin A., Firdaus R.A., Ulfa D.K., Gandidi I.M., Romadhon R. (2022). The immersion cooling technology: Current and future development in energy saving. Alex. Eng. J..

[10] Chen J., Ahmad S., Cai J., Liu H., Lau K.T., Zhao J. (2021). Latest progress on nanotechnology aided boiling heat transfer enhancement: A review. Energy.

[11] Li J.-Q., Gong Z.-Y., Ma H.-R., Wu T., Sun H.-K., Ma Y.-B., Su Z.-L., Kwon J.-T., Wang Y., Li J.-C. (2024). Numerical simulation and thermal analysis of water circulation cooling pipe of pressurized hydrogen ship-board cylinders. Case Stud. Therm. Eng..

[12] Wang J.-X., Li Y.-Z., Zhang Y., Li J.-X., Mao Y.-F., Ning X.-W. (2018). A hybrid cooling system combining self-adaptive single-phase mechanically pumped fluid loop and gravity-immune two-phase spray module. Energy Convers. Manag..

[13] Yu Z.-Q., Li M.-T., Cao B.-Y. (2024). A comprehensive review on microchannel heat sinks for electronics cooling. Int. J. Extrem. Manuf..

[14] Mohammed H.A., Bhaskaran G., Shuaib N.H., Saidur R. (2011). Heat transfer and fluid flow characteristics in microchannels heat exchanger using nanofluids: A review. Renew. Sustain. Energy Rev..

[15] Yang X., Chen J., Qin Y., Wang Y., Wang H., Yu K., Wang Z., Wang M., Wang C. (2025). Synergistic thermo-hydraulic optimization of embedded microchannels: Balancing chip cooling efficiency and pressure fluctuation resistance. Energy.

[16] Zhang N., Jiao B., Ye Y., Kong Y., Du X., Liu R., Cong B., Yu L., Jia S., Jia K. (2022). Embedded cooling method with configurability and replaceability for multi-chip electronic devices. Energy Convers. Manag..

[17] Ye Y., Liu R., Du X., Zhang N., Kong Y., Jiao B., Chen D. (2021). Investigation on multidimensional test vehicle for embedded microfluidic cooling performance evaluation. Appl. Therm. Eng..

[18] Hota S.K., Lee K.-L., Leitherer B., Elias G., Hoeschele G., Rokkam S. (2023). Pulsating heat pipe and embedded heat pipe heat spreaders for modular electronics cooling. Case Stud. Therm. Eng..

[19] Ali N., Srivastava S., Haque I., Yadav J., Alam T., Siddiqui T.U., Siddiqui M.I.H., Cuce E. (2024). Heat dissipation and fluid flow in micro-channel heat sink equipped with semi-elliptical pin-fin structures: A numerical study. Int. Commun. Heat Mass Transf..

[20] Zhao C., Wang G., Deng H., Wang Y. (2026). Multi-objective optimization of heat sinks with micro variable-density pin-fins for hotspot thermal management. Int. J. Heat Fluid Flow.

[21] Alam T., Ansari M.M., Kulakrni K.S., Haque I., Ali N. (2025). Optimization of tapered pin fins for enhanced heat transfer in microchannel heat sink. Int. J. Therm. Sci..

[22] Wang Z., Zheng S., Xu S., Dai Y. (2024). Investigation on the thermal and hydrodynamic performances of a micro-pin fin array heat sink for cooling a multi-chip printed circuit board. Appl. Therm. Eng..

[23] Zhu Q., Chang K., Chen J., Zhang X., Xia H., Zhang H., Wang H., Li H., Jin Y. (2020). Characteristics of heat transfer and fluid flow in microchannel heat sinks with rectangular grooves and different shaped ribs. Alex. Eng. J..

[24] Xu S., Zhang Y., Li Q., Chen X. (2025). Multi-physical field coupling effect in micro pin-fin channel cooling with coaxial-like through-silicon via (TSV) for three-dimensional integrated chip (3D-IC). Appl. Therm. Eng..

[25] Yu B., Lu Z., Wang B., Wang X., Lou J., Yang L., Li W. (2023). A bioinspired programmable Self-Organization approach for designing additively manufactured heat sinks. Energy Convers. Manag..

[26] Zhan S. (2024). Topology optimization of liquid cooling plate for lithium battery heat dissipation based on a bionic leaf-vein structure. Int. J. Heat Mass Transf..

[27] Sadri S., Raveshi M.R., Amiri S. (2012). Efficiency analysis of straight fin with variable heat transfer coefficient and thermal conductivity. J. Mech. Sci. Technol..

[28] Babaelahi M., Raveshi M.R. (2014). Analytical efficiency analysis of aerospace radiating fin, Proceedings of the Institution of Mechanical Engineers. Part C J. Mech. Eng. Sci..

[29] Bouaziz M.N., Aziz A. (2010). Simple and accurate solution for convective–radiative fin with temperature dependent thermal conductivity using double optimal linearization. Energy Convers. Manag..

[30] Alarabi T.H., Mahdy A. (2024). Case study agrivoltaics technology using hybrid, triple magnetized sutterby nanofluid with joule heating application. Case Stud. Therm. Eng..

[31] Babar H., Wu H., Zhang W., Xie Y. (2024). Harnessing nano-synergy: A comprehensive study of thermophysical characteristics of silver, beryllium oxide, and silicon carbide in hybrid nanofluid formulations. J. Mol. Liq..

[32] Raveshi M.R., Keshavarz A., Mojarrad M.S., Amiri S. (2013). Experimental investigation of pool boiling heat transfer enhancement of alumina–water–ethylene glycol nanofluids. Exp. Therm. Fluid Sci..

[33] Zamzamian A., Oskouie S.N., Doosthoseini A., Joneidi A., Pazouki M. (2011). Experimental investigation of forced convective heat transfer coefficient in nanofluids of Al2O3/EG and CuO/EG in a double pipe and plate heat exchangers under turbulent flow. Exp. Therm. Fluid Sci..

[34] Heris S.Z., Shokrgozar M., Poorpharhang S., Shanbedi M., Noie S.H. (2014). Experimental Study of Heat Transfer of a Car Radiator with CuO/Ethylene Glycol-Water as a Coolant. J. Dispers. Sci. Technol..

[35] Japar W.M.A.A., Sidik N.A.C., Saidur R., Asako Y., Yusof S.N.A. (2020). A review of passive methods in microchannel heat sink application through advanced geometric structure and nanofluids: Current advancements and challenges. Nanotechnol. Rev..

[36] Nandakumar V. (2024). Investigating the thermo-physical properties of a new kind of graphitic carbon nitride included ternary hybrid nanofluids and the property correlations. Heliyon.

[37] Lu B., He J. (2024). Equivalent Thermal Conductivity of Topology-Optimized Composite Structure for Three Typical Conductive Heat Transfer Models. Energies.

[38] Xiao W., Lu X., Teng R., Xu Q., Wu J., Xu J., Han Y., Zhou G., Zhan W. (2025). Establishment and simulation study of equivalent model for thermal contact resistance in electronic devices. Can. J. Chem. Eng..

[39] He W., Wang Z., Li J., Li Q. (2023). Investigation of heat transfer performance for through-silicon via embedded in micro pin fins in 3D integrated chips. Int. J. Heat Mass Transf..

[40] Wang R., Qian J., Wei T., Huang H. (2021). Integrated closed cooling system for high-power chips. Case Stud. Therm. Eng..

[41] Razzaq I., Xinhua W., Rasool G., Sun T., Shflot A.S., Malik M.Y., Abbas K., Ali S., Ali A. (2025). Nanofluids for Advanced Applications: A Comprehensive Review on Preparation Methods, Properties, and Environmental Impact. ACS Omega.

[42] Eneren P., Aksoy Y.T., Vetrano M.R. (2022). Experiments on Single-Phase Nanofluid Heat Transfer Mechanisms in Microchannel Heat Sinks: A Review. Energies.

[43] Shit S.P., Pal S., Ghosh N.K., Sau K. (2021). Thermophysical properties of graphene and hexagonal boron nitride nanofluids: A comparative study by molecular dynamics. J. Mol. Struct..

